# Advances in nucleic acid-based cancer vaccines

**DOI:** 10.1186/s12929-024-01102-w

**Published:** 2025-01-21

**Authors:** Hung-Chun Liao, Shih-Jen Liu

**Affiliations:** 1 National Institute of Infectious Diseases and Vaccinology, National Health Research Institutes, Miaoli, 35053 Taiwan; 2 Graduate Institute of Biomedical Sciences, China Medical University, Taichung, 406040 Taiwan; 3 Graduate Institute of Medicine, College of Medicine, Kaohsiung Medical University, Kaohsiung, 307378 Taiwan

**Keywords:** DNA vaccine, mRNA vaccine, Cancer vaccine, Neoantigen

## Abstract

Nucleic acid vaccines have emerged as crucial advancements in vaccine technology, particularly highlighted by the global response to the COVID-19 pandemic. The widespread administration of mRNA vaccines against COVID-19 to billions globally marks a significant milestone. Furthermore, the approval of an mRNA vaccine for Respiratory Syncytial Virus (RSV) this year underscores the versatility of this technology. In oncology, the combination of mRNA vaccine encoding neoantigens and immune checkpoint inhibitors (ICIs) has shown remarkable efficacy in eliciting protective responses against diseases like melanoma and pancreatic cancer. Although the use of a COVID-19 DNA vaccine has been limited to India, the inherent stability at room temperature and cost-effectiveness of DNA vaccines present a viable option that could benefit developing countries. These advantages may help DNA vaccines address some of the challenges associated with mRNA vaccines. Currently, several trials are exploring the use of DNA-encoded neoantigens in combination with ICIs across various cancer types. These studies highlight the promising role of nucleic acid-based vaccines as the next generation of immunotherapeutic agents in cancer treatment. This review will delve into the recent advancements and current developmental status of both mRNA and DNA-based cancer vaccines.

## Introduction

The exploration of cancer treatment has undergone significant transformations over the past few decades, evolving from traditional methodologies to more advanced and targeted approaches. Conventional treatments, such as surgery, chemotherapy, and radiation therapy, have been foundational in cancer management but often have limitations and adverse side effects, highlighting the pressing need for more precise and less harmful options. This necessity has paved the way for the emergence of cancer immunotherapy, a revolutionary approach that leverages the body's innate immune system to fight cancer. Unlike conventional treatments that directly target cancer cells, immunotherapy seeks to support the immune system, enabling it to detect, target, and eradicate cancerous cells effectively.

Cancer immunotherapy encompasses various strategies, including ICIs, adoptive cell transfers (ACTs), oncolytic viral therapy, antibody therapies, cytokine therapies, and cancer vaccines. Immune checkpoint inhibitors, targeting molecules such as CTLA-4 and PD-1/PD-L1, serve to release the brakes on the immune system, allowing it to recognize and attack cancer cells vigorously [1]. Adoptive cell transfer enhances the ability of a patient's autologous immune cells to combat cancer more effectively [2]. Oncolytic viral therapy, a new cancer treatment, uses modified viruses to infect and lyse cancer cells, releasing antigens and cytokines that boost the immune response and inflammation at the tumor site [3]. Cytokines are used to stimulate the immune system by initiating cell signaling. Interferons and interleukins are examples of cytokines used in cancer immunotherapy [4]. Moreover, cancer vaccines represent a proactive approach, intending to prime the immune system against specific tumor antigens, thus preventing cancer development or recurrence.

Among the innovative strategies in cancer immunotherapy, therapeutic cancer vaccines have shown considerable promise in the past decade. Therapeutic cancer vaccines are designed to introduce cancer-specific antigens to a patient’s immune system and aim to elicit specific immune responses against cancer cells, establish long-lasting antitumor memory, and minimize adverse events. These vaccines can be classified into various types based on different tumor antigen platforms, which include (i) peptide/protein vaccines, (ii) whole tumor vaccines, (iii) cell-based vaccines, (iv) viral/bacterial-based vaccines, and (v) nucleic acid-based vaccines. Advancements in next-generation sequencing technology and whole-genome mapping have facilitated the identification of neoantigens in individual tumors [5]. Nucleic acid vaccines have regained substantial attention because of their precision, versatility, and easy production, giving them potential for personalized cancer vaccine development.

This review article seeks to delve into the advancements in nucleic acid-based cancer vaccines, offering a comprehensive overview of the current landscape, including strategies employed, clinical trials underway, and the comparative advantages of nucleic acid vaccines over other vaccine platforms. As the field of cancer immunotherapy continues to expand, nucleic acid vaccines have emerged as a beacon of hope, potentially revolutionizing the approach to cancer treatment and marking a significant step forward in the quest to reinforce the immune system's full therapeutic potential against cancer.

## An overview of nucleic acid-based vaccines

### The features of nucleic acid vaccines

Therapeutic cancer vaccines represent a pioneering front in the battle against cancer, intending to harness the immune system's ability to recognize, attack, and remember cancer cells. Cancer vaccines are broadly categorized based on the type of antigen delivery or the source of antigens used to elicit an immune response [6]. Nucleic acid-based vaccines, including DNA and RNA vaccines, represent a revolutionary approach to vaccination technology. These vaccines introduce genetic material encoding specific tumor antigens into host cells, where the antigens are synthesized and presented to the immune system. Unlike other cancer vaccine platforms, nucleic acid-based vaccines carry unique features that allow rapid vaccine development, manufacturing, and easy customization, making them particularly suited for personalized cancer treatment [7].

#### Precision and specificity

One of the hallmark features of nucleic acid vaccines is their precision and specificity. These vaccines are designed to encode specific antigens expressed by cancer cells, enabling the immune system to target and eliminate cancer cells more effectively. Unlike whole tumor cell vaccines, which present a broad array of antigens to the immune system, nucleic acid vaccines can be tailored to express only the most relevant and mutated antigens to generate a robust immune response. This specificity reduces the risk of autoimmunity and enhances the efficacy of the vaccine [8, 9].

#### Rapid development and scalability

The development process for nucleic acid vaccines, particularly DNA and mRNA-based platforms, is significantly faster and more scalable than traditional vaccine platforms, such as protein- or viral vector-based vaccines. One key advantage is that once the genetic sequence of a target antigen is identified, nucleic acid vaccines can be rapidly designed and synthesized without the need for complex cell culture systems. This rapid design process allows for the quick adaptation of vaccines to new or mutating cancer antigens, which is crucial in personalized cancer vaccines[10]. Additionally, the production of nucleic acid vaccines is relatively straightforward, relying on well-established in vitro transcription (IVT) and plasmid DNA manufacturing processes. These methods are easily scalable, enabling mass production at an industrial level, which is particularly advantageous in the face of new cancer targets or widespread needs [11].

In contrast, protein-based vaccines require more time-consuming steps, including the expression of recombinant proteins in mammalian or bacterial systems, followed by laborious purification processes [12]. The costs and time associated with these steps make protein vaccines less suitable for rapid responses, especially when dealing with the personalized nature of many cancer vaccines [13]. The scalability and speed of production of nucleic acid vaccines are particularly advantageous for responding quickly to emerging cancer targets and personalized cancer vaccine approaches.

#### Safety profile

Nucleic acid vaccines, including DNA and mRNA vaccines, have demonstrated a favorable safety profile, a critical aspect distinguishing them from traditional vaccines and viral vector platforms. Viral vector vaccines, which utilize live-attenuated or replication-deficient viruses, present potential risks such as viral reactivation, immunogenicity to viral components, or genome integration, which could theoretically lead to mutagenesis or oncogenesis [14, 15]. In contrast, nucleic acid vaccines are noninfectious, minimizing the risk of unintended infection.

DNA vaccines remain episomal, meaning they do not integrate into the host genome but exist as separate genetic material within the nucleus [16, 17]. This episomal nature eliminates concerns of genomic instability or insertional mutagenesis. Furthermore, studies have shown that mRNA vaccines are transient and designed to degrade naturally after protein expression [18]. Once the mRNA is translated, cellular ribonucleases degrade the mRNA, further reducing the likelihood of long-term safety issues. Additionally, mRNA does not require nuclear entry, thus avoiding the risks associated with nuclear localization.

This favorable safety profile makes nucleic acid vaccines particularly advantageous for cancer immunotherapy, where patients often have compromised immune systems or undergo immunosuppressive treatments [19]. The reduced risk of autoimmunity and the ability to avoid pre-existing immunity to viral vectors further support their use in a clinical oncology setting.

#### Induction of broad immune responses

Nucleic acid vaccines have demonstrated the capability to induce robust and broad-spectrum immune responses. The endogenous production of the antigens encoded by these vaccines allows for presentation via major histocompatibility complex (MHC) class I and II pathways, leading to the activation of both CD8^+^ cytotoxic T lymphocytes (CTLs) and CD4^+^ helper T cells [20, 21]. This dual activation is crucial for an effective anti-tumor immune response, as CD8^+^ T cells are responsible for directly killing cancer cells [22], while CD4^+^ T cells support the proliferation and activation of CD8^+^ T cells and B cells [23]. Furthermore, nucleic acid vaccines have been shown to induce the production of high-affinity neutralizing antibodies [24].

#### Flexibility and personalization

The flexibility of nucleic acid vaccines to encode virtually any antigen of interest makes them particularly suited for personalized cancer vaccine approaches. These vaccines can be rapidly designed and synthesized to incorporate multiple tumor-specific antigens. With advances in next-generation sequencing (NGS) and tumor profiling technologies, vaccines can be customized to target mutated proteins unique to individual cancers that arise from somatic mutations. This approach enables the development of highly personalized therapeutic strategies to elicit robust, targeted immune responses against cancer cells, minimizing off-target effects and potential immune-related toxicity [25, 26]. Furthermore, nucleic acid vaccines can be produced more quickly than traditional peptide or protein-based vaccines, requiring more labor-intensive processes such as protein expression and purification [27]. This rapid adaptability makes nucleic acid-based platforms ideal for cancers with high mutational burdens, where tumor antigens are constantly evolving. Thus, the nature of nucleic acid vaccines positions them as a promising tool offering new hope for tailoring immunotherapy to individual patient needs [28].

In conclusion, nucleic acid-based vaccines significantly advance cancer immunotherapy, offering unparalleled specificity, rapid development, safety, and the potential for personalization. Their ability to induce broad immune responses positions them as a highly effective tool in the fight against cancer. As research progresses and technological hurdles are overcome, nucleic acid vaccines are poised to transform cancer treatment paradigms.

### Molecular mechanisms of action of nucleic acid vaccines

**Fig. 1:**
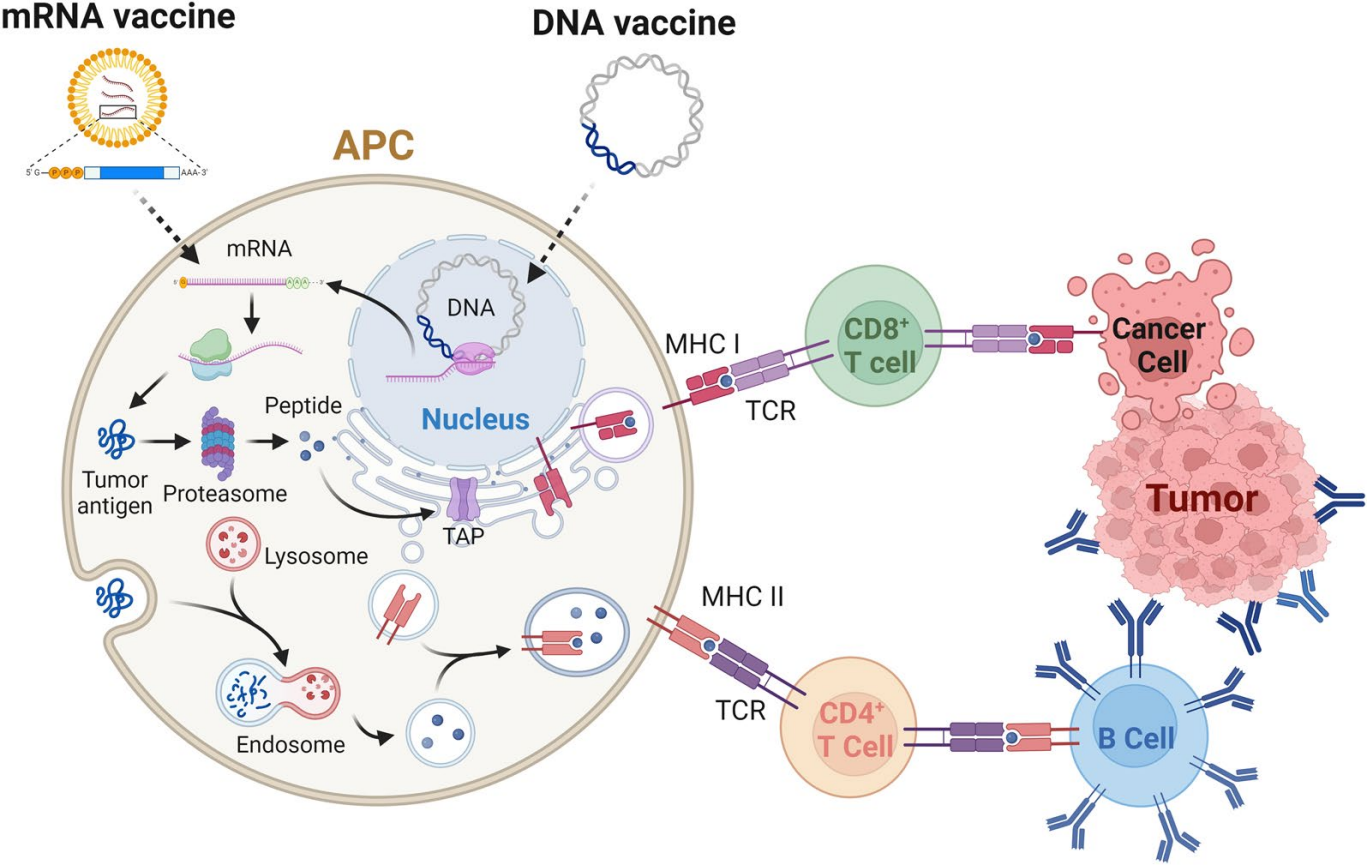
Molecular mechanisms of DNA and mRNA cancer vaccines. DNA is delivered to the cell nucleus and then transcribed to mRNA. The mRNA is translated to protein in the cytosol, and the protein is degraded via the proteasome into small peptides. These peptides are translated into the ER and bind to MHC class I molecules after fine-tuning. MHC class I/peptide complexes are present in CD8^+^ T cells to kill cancer cells. However, the mRNA was delivered into the cell cytosol and translated to the protein through the same mechanisms that activate CD8^+^ T cells. Alternatively, the extracellular proteins expressed by DNA or mRNA vaccines can be taken up by APCs and degraded by the endosome-lysosome pathway, which then binds to MHC class II molecules to present to CD4^+^ T cells. Activated CD4^+^ T cells induce antigen-specific B cells to secrete antibodies targeting tumor cells. In summary, DNA and mRNA cancer vaccines can induce both CD4^+^ and CD8^+^ T cells to clear cancer cells. (Figure created with Biorender)

The mechanism of action of these vaccines can be understood through a series of steps: uptake of nucleic acid into host cells, antigen production, processing, and, ultimately, antigen presentation to the immune system (Fig. 1).

#### DNA vaccines

The process of vaccine uptake may occur via natural diffusion across transient plasma membrane pores, receptor-mediated endocytosis, caveolar endocytosis, or even through membrane disruptions caused by mechanical forces. After entering cells, these plasmids travel to the nucleus and serve as templates for transcription. The transcribed mRNA is then translated into proteins or antigens using the machinery of the host cell. These antigens undergo posttranslational modifications similar to those of naturally occurring proteins and are presented on the cell surface by major histocompatibility complex (MHC) molecules [29]. In somatic cells, antigenic peptides are presented to CD8^+^ T cells via MHC class I molecules [30]. Proteins either secreted or released into the extracellular matrix are then captured by antigen-presenting cells (APCs) and displayed via MHC class II molecules to CD4^+^ T helper cells, which, upon activation through costimulatory signals, promote cellular or humoral immune responses based on their subtype (Th1, Th2) [31]. Secreted antigens also provoke the production of antibodies and B-cell responses, enhancing both T-cell- and antibody-mediated immunity [21].

Additionally, the intrinsic components of plasmid DNA can trigger the innate immune system. Intracellular sensors that recognize the double-stranded structure of DNA contribute to overall immunogenicity [32–34]. For example, STING plays a crucial role in detecting plasmid DNA and initiating immune responses. Deficiencies in STING, for instance, have been shown to impair adaptive immune responses in DNA vaccine recipients [35]. Moreover, various cytosolic and endosomal proteins, including DDX41, RIG1, and AIM2, interact with B-form DNA, contributing to the immune response by activating inflammatory pathways [36].

Moreover, innate immune responses are activated through pattern recognition receptors (PRRs), including Toll-like receptors (TLRs) that recognize pathogen-associated molecular patterns (PAMPs). CpG motifs within plasmid DNA, uncommon in vertebrate genomes but prevalent in bacteria, serve as danger signals that activate TLR9 within the endocytic pathways of APCs [37]. This interaction initiates a signaling cascade involving MYD88, IRAK, TRAF6, and NF-κB, leading to the release of inflammatory cytokines and chemokines [38]. This cascade ultimately enhances dendritic cell-mediated Th1 immune responses by producing IL-12 and IFN-α [39]. This cascade also facilitates NK cell activation and  CD8^+^ T-cell proliferation. Conversely, methylated CpG-rich bacterial DNA has been noted for its immune-suppressive properties [37], highlighting the complexity of DNA vaccine interactions within the immune system.

#### mRNA vaccines

The mechanism of mRNA vaccines parallels that of their DNA counterparts in the initial steps but diverges because mRNA can be synthesized by in vitro transcription (IVT). This process generates synthetic mRNA that mimics fully mature native mRNA present in the eukaryotic cytosol [40]. The synthetic mature mRNA encodes the protein(s) of interest in an open reading frame (ORF) flanked by untranslated regions (UTRs) and ideally consists of a 5’ cap and a poly(A) tail. Upon administration, mRNA vaccines deliver the mRNA directly to the cytoplasm, bypassing the need for nuclear entry and allowing for swift translation of the mRNA into proteins by ribosomes. This quick translation leads to the immediate production of antigenic proteins, which undergo posttranslational modifications, folding, and presentation to the immune system—mirroring the protein synthesis seen in RNA virus infections and their resultant immune responses.

Once administered, the exogenous mRNA and its associated delivery components are detected as foreign by pattern recognition receptors (PRRs) located on cell membranes, in endosomes, and within the cytoplasm in host cells. These receptors include retinoic acid-inducible gene I (RIG-I), anti-melanoma differentiation-associated gene 5 (MDA5), and LGP2, predominantly expressed on APCs. This recognition is crucial in initiating the intrinsic immune response [41]. The immunogenic potential of IVT mRNA is mediated primarily by TLR7 and TLR8. TLR7, found on B cells, macrophages, and dendritic cells (DCs), facilitates the detection of single-stranded RNA (ssRNA) [42]. Activation through the MYD88-dependent TLR7 pathway stimulates B cells, enhances the production of proinflammatory cytokines, and improves antigen presentation and memory B-cell survival.

Furthermore, the MYD88 pathway enhances type I interferon (IFN I) responses and promotes a proinflammatory state through additional cytokine production. When the mRNA synthesis process introduces double-stranded RNA (dsRNA), it can further activate intracellular sensors such as protein kinase R (PKR) and oligoadenylate synthetase (OAS) [43], leading to mRNA degradation mediated by the IFN I response [44]. The collective activation of these PRRs and the resulting IFN I production can have mixed effects, potentially beneficial or detrimental, in the context of anticancer immunotherapy, depending on the balance between immune activation and inflammation.

## Advances in DNA-based cancer vaccines

### Historical perspective of DNA vaccines

The concept of DNA vaccines originated in the 1960s with the discovery that naked DNA can be used to transfect mammalian cells in vivo [45]. Subsequent research demonstrated that this transfection could lead to antigen-specific antibody responses and CTL responses [46], laying the foundation for the use of DNA vaccines as novel vaccine platforms. In 1998, the first human phase I clinical trial of a DNA vaccine for HIV-1 infection showed specific immune responses but inconclusive efficacy [47]. Subsequent trials with DNA vaccines encoding malaria proteins also demonstrated specific immune responses in healthy volunteers [48]. Despite these early successes, DNA vaccines face challenges in translating results from animal models to humans, with low immunogenicity being a significant hurdle.

Over the years, various optimization strategies have been developed to enhance the immunogenicity of DNA vaccines. While DNA vaccines have been more successful in veterinary use, with licensed products against infectious diseases, cancer immunotherapy, and gene therapy applications [49], their application in humans has been limited. Despite these challenges, DNA vaccines have shown promise in clinical trials for infectious diseases such as Ebola virus [50], Zika virus [51], and Middle East respiratory syndrome (MERS)[52]. The COVID-19 pandemic also led to significant progress in DNA vaccine development, with the approval of the first human DNA vaccine, ZyCoV-D, in 2021 [53]. Additionally, double-stranded (ds)DNA is more stable than single-stranded (ss) mRNA and is prone to rapid degradation in vivo. Therefore, while mRNA vaccines require stringent cold chain storage conditions, which could limit their distribution and accessibility, technological advancements are underway to improve their thermal stability. Conversely, DNA vaccines tend to be more stable at a range of temperatures, offering a logistical advantage in certain settings (Table 1).

**Table 1:** Comparing DNA and mRNA vaccines for cancer immunotherapy

Attribute	DNA vaccines	mRNA vaccines
Stability	Generally stable at 2–8 °C, can be freeze-dried for easier transport and longer shelf life	Requires ultracold storage (− 20 °C to − 70 °C) to maintain stability, complicating logistics
Delivery	Requires delivery to the nucleus, which can be less efficient	Only needs to reach the cytoplasm, making delivery potentially more efficient
Delivery System	Typically delivered via viral vectors, electroporation, or gene gun	Often encapsulated in lipid nanoparticles (LNPs) to enhance stability and delivery
Production	Relatively easy to produce in large quantities and at a low cost	Production is fast but can be initially more expensive; scale-up has been increasingly optimized
Mechanism	Host cells uptake DNA, which is then transcribed into mRNA and translated into proteins	Host cells translate mRNA into protein directly
Immune Response	Induce a strong cellular and humoral immune response due to the transcription and translation processes in the host cells	Potent inducer of both cellular and humoral immune responses, especially effective at stimulating helper and cytotoxic T cells
Adjuvant Requirement	Often requires adjuvants to enhance immunogenicity	Often includes innate immunostimulatory properties and may not require additional adjuvants
Safety	Long-term expression might risk integration into host DNA, although this is very rare with modern vectors	No risk of integration into the host genome. Degraded by normal cellular processes

### Improving the immunogenicity of DNA vaccines

**Fig. 2:**
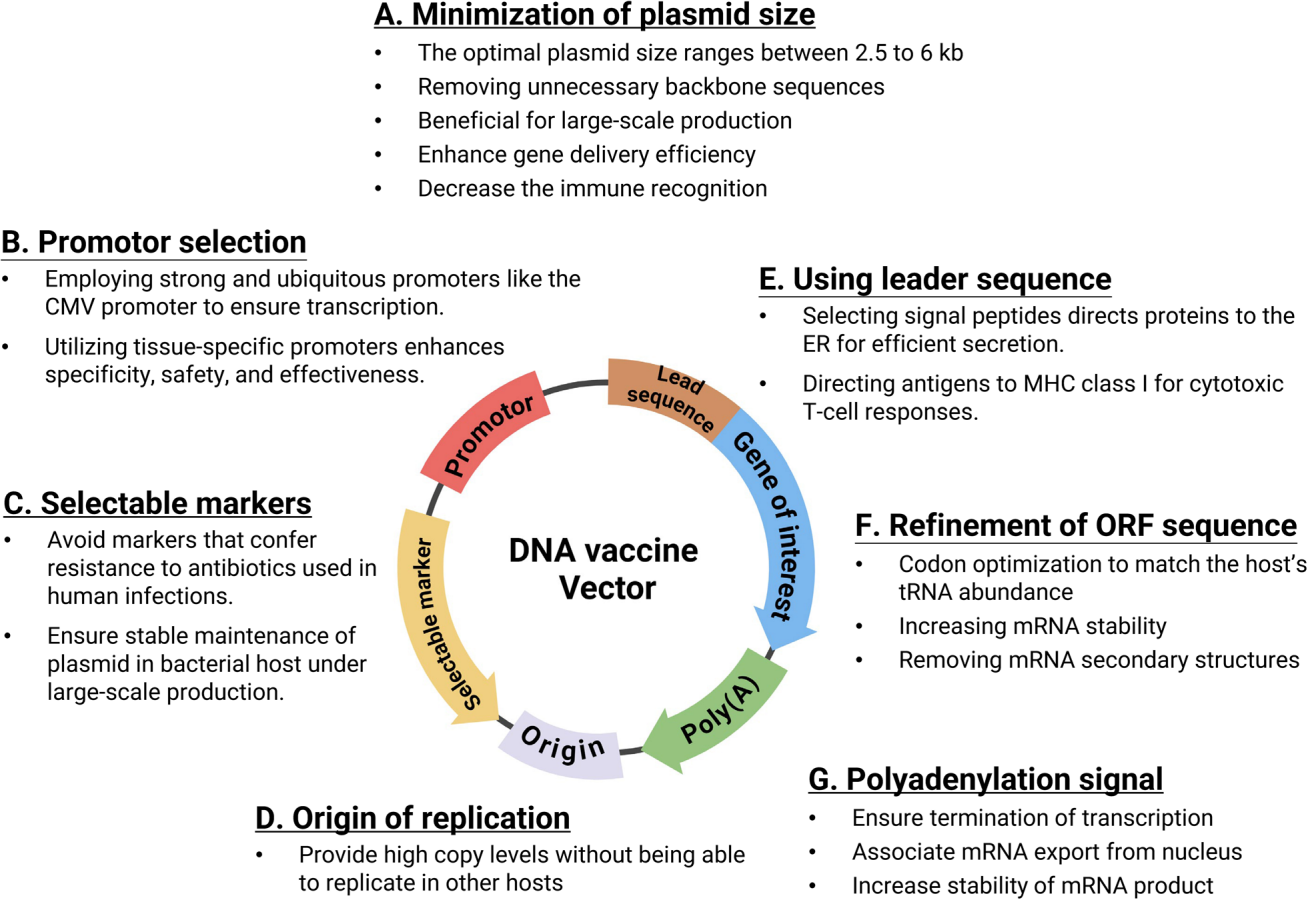
Key Design Elements for an Optimized DNA Vaccine Vector. Many factors need to be considered when designing a DNA vaccine. These elements collectively aim to optimize DNA vaccines for efficacy and safety. **A** Minimization of Plasmid Size: This involves removing unnecessary backbone sequences to facilitate large-scale production, enhance gene delivery efficiency, and decrease immune recognition. **B** Promoter Selection: The use of strong and ubiquitous promoters ensures transcription. Tissue-specific promoters are employed to enhance specificity, safety, and effectiveness. **C** Selectable Markers: Avoid markers that confer resistance to antibiotics used in human infections and ensure stable maintenance of the plasmid in bacterial hosts during large-scale production. **D** Origin of replication: providing high copy numbers of the plasmid without enabling replication in other hosts. **E** Using the leader sequence, signal peptides that direct proteins to the ER for efficient secretion and target antigens to MHC class I for cytotoxic T-cell responses. **F** Refinement of the ORF sequence: codon optimization to match the host tRNA abundance, increasing mRNA stability and removing mRNA secondary structures. **G** Polyadenylation signal: This signal ensures the termination of transcription, promotes mRNA export from the nucleus and increases the stability of the mRNA product. (Figure created with Biorender)

A fundamental limitation of DNA vaccines is their inconsistent ability to elicit strong immune responses, primarily due to suboptimal DNA transfection and immunostimulation. The efficacy of DNA transfection varies significantly because the complex structures of cellular and nuclear membranes differ among individuals. For successful immunization, plasmids must traverse the phospholipid-rich cell membrane via pinocytosis or endocytosis. Additionally, these plasmids need to avoid degradation by lysosomes, endosomes, and nucleases. Along with physical and chemical delivery techniques to overcome these obstacles, using immunomodulatory elements in DNA vaccines has helped increase immunogenicity (Fig. 2).

#### Conventional adjuvants

Conventional adjuvants, including aluminum salts and CpG motifs, act as immune stimulators and vaccine delivery systems, activating APCs and supplying T-helper signals. The synergistic effect of these adjuvants in DNA vaccines significantly enhances the presentation of tumor antigens and the priming of T and B cells, resulting in a robust and durable antitumor-immune response.

When used as an adjuvant for DNA vaccines, aluminum salts form positively charged aggregates in physiological pH buffers, which can adsorb plasmid DNA. [54, 55]. Although these aggregates do not significantly enhance the expression of the encoded antigen [56], they effectively prolong the interaction of the antigen with immune cells at the injection site. This extended interaction facilitates the uptake and presentation of the antigen by APCs through phagocytosis. The activation of the inflammasome complex by aluminum salt aggregates induces the production of proinflammatory cytokines, promoting a predominantly Th2 antibody response. When combined with the inherent Th1 response elicited by plasmid DNA in DNA vaccines, this leads to a balanced Th1/Th2 immune response, enhancing overall vaccine immunogenicity [57].

However, DNA vaccines possess adjuvant activity owing to the presence of unmethylated CpG dinucleotides in particular base contexts (CpG-S motifs). Coadministering CpG ODN with suboptimal amounts of a DNA vaccine also boosts the immune response by enhancing cytokine and immunoglobulin secretion. CpG ODN also promotes the upregulation of MHC and costimulatory molecules on the surface of immune cells and directly activates monocytes, macrophages, and dendritic cells to secrete a range of cytokines and chemokines. This leads to a Th1-biased cytokine production pattern, predominantly involving IL-12 and IFN-γ, fostering strong CTL responses, which are crucial for effective DNA vaccination [58].

#### Assistant delivery systems

Innovative approaches to the delivery of DNA vaccines aim to enhance their immunogenicity through various methods that optimize the cellular uptake of the vaccine. One technique involves the temporary disruption of host cell plasma membranes through mechanical means such as needle-induced punctures or hydrostatic stress, which creates large transient openings that facilitate the entry of DNA plasmids. This active disruption has significantly boosted DNA expression in diverse tissues, including muscle myofibers and hepatocytes, leading to a more robust immune response. Some agents that cause muscle fiber destruction and subsequent muscle regeneration, such as bupivacaine and cardiotoxin, have been used in DNA vaccination [59]. However, destroying muscle tissues to improve DNA vaccine efficacy may not be feasible in humans. The further observation that in vivo electroporation can dramatically enhance gene expression by 10–1000-fold has led to great interest in assessing the ability of this delivery method to improve the immunogenicity and effectiveness of DNA vaccines [60].

DNA electroporation in vivo was first tested in 1999 in an independent study and showed successful results [61, 62]. DNA intramuscular injection following electrotransfer has been shown to efficiently deliver DNA plasmids into tissues due to the permeabilization of cell membranes and DNA electrophoresis [63, 64]. DNA electroporation is a promising technique that can express a large amount of antigen and induce more robust immune responses. However, the major challenge is the application of electric devices to humans. Fortunately, the first human clinical trial using the IL-12 gene was initiated in the U.S. by the company Inovio Biomedicals to treat melanoma [65]. Many preclinical and clinical studies on cancer treatment have been carried out over the past 10 years, and the optimization of protocols and improvement of electroporation procedures are ongoing [66]. Recently, the most effective delivery methods using two-needle electrodes for electroporation with an electroporator have been developed [67]. The electroporation conditions were bridged to a clinically used electroacupuncture machine and for clinical trials [68]. In addition to electroporation, several alternative delivery systems have been developed, such as microneedle arrays [69], jet injectors [70–73], the gene gun method [74–76], and suction-based devices [77–80]. However, the efficacy still needs to be improved.

Recently, mRNA vaccine technology has been used worldwide because of the COVID-19 pandemic. One of the significant improvements in mRNA vaccines is the use of 4-component lipid nanoparticles (LNPs), which contain an ionizable lipid that can efficiently enhance mRNA release in the cytosol. Based on this observation, the DNA vaccines encoding spike protein were also tested for the formulations with LNPs. Our studies showed that the LNP-encapsulated DNA vaccine encoding the trimerization spike protein of SARS-CoV-2 induced robust humoral responses, which are comparable to those induced by the mRNA vaccine with LNP delivery in animals [81]. After optimizing the LNP formulation, DNA/LNPs could efficiently induce protective immunity against the SARS-CoV-2 challenge comparable to the BioNTECT/Pfizer COVID-19 mRNA vaccine [82]. Furthermore, an amphiphilic bioresorbable copolymer (ABC), PLA-PEG, was reported as a surface engineering agent that endows LNPs with stability during preparation and biocompatibility postvaccination [83]. These results indicated that the DNA vaccines delivered by the formulation with LNPs could be a potential platform for emerging infectious disease outbreaks.

These technologies not only improve the distribution and uptake of DNA vaccines at the cellular level but also offer significant practical benefits, such as reducing pain and eliminating the risks associated with traditional needle injections, enhancing patient compliance, and widening the applicability of DNA-based immunization strategies.

#### Encoding immunomodulators

To augment the immunogenicity of DNA vaccines, particularly in combating tumor-induced immune resistance, innovative strategies include encoding immunomodulatory molecules alongside the target antigen within the same or separate plasmids. Tumors often evade immune surveillance by downregulating MHC molecules [84] and upregulating immunosuppressive cytokines such as TGF-β and IL-10 [85]. By incorporating genes for stimulatory cytokines, chemokines, and signaling molecules, these advanced DNA vaccines can counteract the immunosuppressive environment of tumors [86, 87].

Cytokines such as interleukin-2 (IL-2) [88, 89] and granulocyte–macrophage colony-stimulating factor (GM-CSF) [90–92] are frequently encoded by these vaccines due to their potent ability to enhance cellular immune responses. IL-2 promotes the activation and proliferation of T cells and natural killer (NK) cells, while GM-CSF boosts the recruitment and maturation of dendritic cells at the vaccination site, facilitating more effective antigen presentation. The localized expression of these cytokines at the vaccination site not only reduces the potential systemic toxicity associated with their widespread administration but also ensures a concentrated immune response where needed [93].

Additionally, chemokines such as CXCL10 [94] and CCL21 [95] enhance immune cell trafficking to the vaccine site, improving the recruitment and activation of various immune cells. These chemokines aid in orchestrating a targeted immune attack on the tumor by guiding immune effector cells to the tumor microenvironment (TME) [96].

Moreover, the integration of costimulatory molecules such as CD80 (B7.1) and CD86 (B7.2) into vaccine design plays a crucial role in amplifying T-cell responses [97]. These molecules enhance the interaction between T cells and antigen-presenting cells, a critical step in achieving a robust and effective immune response. The inclusion of CD40, another member of the TNF superfamily, in DNA vaccination further aids in the maturation of dendritic cells and the induction of cytotoxic T-cell responses, which are essential for effective antitumor immunity [98].

#### Facilitating antigen processing and presentation

Antigens encoded by plasmid DNA can be directly introduced into antigen-presenting cells, which undergo proteasomal degradation to form small peptide fragments. These peptides are then transported into the endoplasmic reticulum (ER) via transporters associated with antigen processing (TAP), where they are refined and loaded onto MHC class I molecules. The resulting peptide/MHC class I complexes are then presented on the cell surface to activate CD8^+^ T cells [29]. Enhancing the efficiency of antigen presentation via MHC class I is pivotal for boosting CD8^+^ T-cell activation[99]. Various methods to augment MHC class I presentation have been explored in animal models and clinical trials (Fig. 1).

For instance, fusing DNA-encoded antigens with ubiquitin (Ub) molecules increases proteasome degradation. Ubiquitin, crucial for degrading intracellular polypeptides, attaches to a lysine residue on a protein, forming a poly-Ub chain that targets proteins for proteasome degradation. It has been shown that immunization with Ub antigens enhances CTL responses, increases IFN-γ secretion, and improves protection or therapeutic outcomes. [100–103]. Additionally, fusing the antigen with the translocation domain (domain II) of *Pseudomonas aeruginosa* exotoxin A [104] enhances proteasome-mediated degradation and MHC class I presentation to CD8^+^ T cells.

Another strategy to enhance the CTL response focuses on improving MHC class I and peptide assembly within the ER. Calreticulin (CRT), a prominent Ca2 + -binding protein in the ER, assists in peptide handling within this organelle. [105]. The linkage of CRT to the human papillomavirus type-16 (HPV-16) E7 (CRT/E7) DNA vaccine can dramatically enhance CTL responses [106]. In the presence of the ER stress-inducing agent 3-bromopyruvate (3-BrPA), CRT/E7 can further increase CD8^+^ T-cell responses and antitumor effects [107]. The CRT/E7 DNA vaccine also efficiently inhibited HPV16 E6/E7-expressing oral tumors in HLA-A2 transgenic mice [108]. Clinical trials exploring the efficacy of CRT/E7 DNA vaccines are currently underway.

The second approach for ER targeting is the linkage of the ER-targeting signal peptide (MLLPVPLLLGLLGLAAAL, SP) and/or the ER-retention sequence KDEL to mutated HPV E6 and E7 (SP‑E6E7m‑KDEL) to immunize mice, which can induce potent antitumor immunity [109]. In addition to the endogenous ER-targeting sequences, the exogenous ER-targeting sequence derived from the adenovirus E3 leader sequence (E3/19 K) has been demonstrated to facilitate epitope transport into the ER and, therefore, enhance specific immunity [110, 111]. The E3/19 K sequence has been linked with the tumor-associated antigen L6 (TAL6) to design a DNA cancer vaccine. The E3/19 K-fused TAL6 DNA vaccine can induce protective effects on EL4 thymoma and melanoma models [112]. B-cell-activating factor (BAFF) is secreted through the ER–Golgi apparatus pathway. Fusing E7 to BAFF could enhance the presentation of MHC class I molecules and the antitumor effects of DNA vaccines [113]. These approaches could enhance antigen processing and presentation by MHC class I molecules to CTLs, which is critical for inducing efficient antitumor effects.

#### Optimized prime-boost strategies

The "prime-boost" strategy starts with the administration of a DNA vaccine as the prime to introduce cancer-specific antigens. Subsequent booster vaccinations can employ the same DNA vaccine (homologous boost) or a different type of vaccine, such as viral or bacterial vectors (heterologous boost) encoding the same or different antigens. This strategic variation in vaccine delivery has been shown to enhance the induction of antigen-specific T-cell responses significantly [114] and facilitate the differentiation of memory T cells [115], providing a strong, durable immune defense against tumor cells [116]. Research indicates that heterologous prime-boost regimens, particularly, can generate more potent cell-mediated immune responses, effectively overcoming the weaker T-cell activation often associated with homologous boosting [117]. By repeatedly stimulating the immune system, the prime-boost approach intensifies the initial immune reaction. It ensures long-term cellular immunity for cancer surveillance and protection [118].

### Clinical studies of DNA cancer vaccines

A review of clinical trials focusing on DNA vaccines against cancers before 2019 revealed that these vaccines predominantly encoded tumor-associated antigens (TAAs). TAAs are present in normal cells but are primarily expressed in tumor cells, making them strategic targets for inducing antitumor immune responses. These antigens fall into several categories, including overexpressed proteins such as MUC1, TAL6, survivin, Ep-CAM, WT1, and Her2/Neu; differentiation antigens such as prostate-specific antigen and tyrosinase; and cancer/testis antigens such as NY-ESO-1 and the MAGE family [119].

Several ongoing clinical trials are evaluating the efficacy of DNA-based cancer vaccines that utilize nonmutated tumor antigens to combat various cancers. The most extensively studied cancers in these trials are breast, prostate, and cervical cancers (Table 2). Typically, these vaccines target well-known TAAs such as Mam-A or HER2 in breast cancer, prostatic acid phosphatase (PAP) in prostate cancer, and oncoviral antigens such as the E6/7 HPV protein in cervical cancer [120].

**Table 2:** DNA vaccines for cancer immunotherapy in clinical trial

Collaborator/ Sponsor	Brand	Encoding	Combination therapy	DNA delivery	Cancer type	Start	Phase	NCT	Status
University of Wisconsin, Madison	pTGV-HP	prostatic acid phosphatase (PAP)	rhGM-CSF	ID	Prostate cancer	2011	II	NCT01341652	Completed
			Nivolumab (a-PD1 Ab), GM-CSF	ID	Prostate cancer	2018	II	NCT03600350	Active
	pTGV-AR	Androgen Receptor Ligand-Binding Domain (AR LBD)	GM-CSF	ID	Prostate cancer	2015	I	NCT02411786	Completed
			Degarelix, Nivolumab	ID	Prostate cancer	2021	I/II	NCT04989946	Recruiting
	pTGV-HP + pTGV-AR	PAP + AR LBD	Pembrolizumab (a-PD1 Ab)	ID	Castration-resistant Prostate cancer Metastatic cancer Prostate cancer	2019	II	NCT04090528	Recruiting
University of Washington	**WOKVAC:** pUMVC3-IGFBP2-HER2-IGF1R	IGFBP2, HER2, and insulin-like growth factor 1 receptor precursor (IGF-1R)	Carboplatin, Paclitaxel	ID	Ovarian cancer	2017	II	NCT03029611	Terminated
			rhu GM-CSF (Sargramostim)	ID	Breast cancer	2016	I	NCT02780401	Active
			Paclitaxel and Trastuzumab Pertuzumab	ID	Breast cancer	2022	II	NCT04329065	Recruiting
	**STEMVAC:** pUMVC3-CD105/Yb-1/SOX2/CDH3/ MDM2-polyepitope:	CD105, Y-box binding protein-1, SRY-box 2, cadherin 3, murine double minute 2	/	ID	Breast cancer	2015	I	NCT02157051	Active, not recruiting
			rhu GM-CSF	ID	Breast cancer	2022	II	NCT05455658	Recruiting
				ID	Lung Non-Squamous Non-Small Cell Carcinoma	2023	II	NCT05242965	Recruiting
Washington University School of Medicine	Mammaglobin-A DNA Vaccine	Mam-A		IM EP	Breast cancer	2015	I	NCT02204098	Recruiting
Immunomic Therapeutics	ITI-1001	IE-1, pp65 and gB	LAMP1	IM EP	Glioblastoma	2023	I	NCT05698199	Recruiting
INOVIO	INO-5410	Wilms tumor gene-1 (WT1) antigen, prostate-specific membrane antigen (PSMA) and human telomerase reverse transcriptase (hTERT) genes	Cemiplimab (a-PD-1 Ab), radiation and chemotherapy; INO-9012 (IL-12 DNA)	IM EP	Glioblastoma	2018	I/II	NCT03491683	Active
			INO-9012, Atezolizumab (a-PD-L1 Ab)	IM EP	Urothelial carcinoma	2018	I/II	NCT03502785	Active
	VXG-3100	HPV E6/E7	/	IM EP	Cervical cancer	2017	III	NCT03185013	Completed
			/	IM EP	Anal neoplasm	2018	II	NCT03499795	Completed
			/	IM EP	CIN 2/3	2018	II	NCT01304524	Completed
			/	IM EP	Cervical HSIL	2019	III	NCT03721978	Completed
National Cancer Institute (NCI)	MEDI0457 (INO-3112)	HPV E6/E7	INO-9012, Durvalumab (a-PD-L1 Ab)	IM EP	HPV-16/18 associated cancers	2018	II	NCT03439085	Active, not recruiting
	pNGVL4a CRTE6E7L2	HPV E6/E7/L2		EP	HPV-16 positive cervical neoplasia	2020	I	NCT04131413	Recruiting
Genexine	GX-188E	HPV E6/E7	/	IM	Cervical cancer	2015	II	NCT02596243	Unknown
			GX-I7 encoding IL7 receptor agonist, Imiquimod	IM	Cervical cancer	2017	/	NCT03206138	Unknown
			Pembrolizumab (a-PD1 Ab)	IM EP	Cervical cancer	2018	I/II	NCT03444376	Completed
**Collaborator/ Sponsor**	**Brand**	**Encoding**	**Combination therapy**	**DNA delivery**	**Cancer type**	**Start**	**Phase**	**NCT**	**Status**
Washington University School of Medicine		Personalized polyepitopes	/	IM EP	Triple Negative Breast Cancer	2015	I	NCT02348320	Completed
		Neoantigens	Durvalumab (a-PD-L1 Ab)	IM EP	Triple Negative Breast Cancer	2019	I	NCT03199040	Terminated
		Neoantigens	Darvalumab Tremelimumab (a-CTLA-4 Ab)	IM EP	Renal cell carcinoma	2019	II	NCT03598816	Withdrawn
		Personalized neoantigen	Durvalumab	IM EP	Small Cell Lung Cancer	2022	II	NCT04397003	Recruiting
		Personalized neoantigen	Retifanlimab	IM EP	Unmethylated glioblastoma	2023	I	NCT05743595	Recruiting
		Personalized neoantigen	INO-9012 (IL-12 DNA)	IM EP	Unmethylated glioblastoma	2020	I	NCT04015700	Active, not recruiting
		Personalized neoantigen		IM EP	Pediatric Recurrent Brain Tumor	2024	I	NCT03988283	Not yet recruiting
Bristol-Myers Squibb Bavarian Nordic		Neoantigens	Nivolumab (a-PD-1 Ab), Ipilimumab (a-CTLA-4 Ab) and Prostvac	IM EP	Metastatic Hormone-Sensitive Prostate Cancer	2018	I	NCT03532217	Completed
National Cancer Institute (NCI)		Personalized neoantigen: pING vector + prioritized neoantigens + mesothelin epitopes	Chemotherapy	IM EP	Pancreatic cancer	2018	I	NCT03122106	Terminated
Geneos Therapeutics	GNOS-PV02	Personalized neoantigen	INO-9012 Pembrolizumab	ID EP	HCC	2020	I/II	NCT04251117	Active, not recruiting

Although DNA vaccines have demonstrated potential in delaying tumor growth and generating strong immune responses, particularly antigen-specific CTL responses, they alone may not be sufficient to circumvent the tumor's immune evasion tactics. These tactics include the natural selection of less immunogenic tumor cell clones and the recruitment of immunosuppressive cells to the TME, such as myeloid-derived suppressor cells (MDSCs) and regulatory T cells (Tregs), which can cause effector T-cell exhaustion. To achieve optimal effectiveness, cancer DNA vaccines should ideally be combined with other therapeutic strategies that enhance the antigen response and counteract immunosuppression within the TME [121].

#### Prostate cancer

Two DNA vaccines, pTVG-HP and pTVG-AR, employ electroporation as their delivery strategy and are currently under investigation in clinical trials for their efficacy and safety in treating prostate cancer. The pTVG-HP vaccine targets prostatic acid phosphatase (PAP), an antigen expressed in the majority of prostate cancer cells. Early-phase clinical trials have demonstrated that pTVG-HP is capable of inducing PAP-specific immune responses in patients with prostate cancer [122]. These studies have also shown an acceptable safety profile, with minimal adverse effects reported. A phase II study focused on evaluating the efficacy of combining GM-CSF in patients with recurrent prostate cancer, examining potential improvements in metastasis-free survival and overall therapeutic outcomes [123]. However, a phase II trial conducted to evaluate vaccination with PD-1 blockade, using nivolumab, in patients with recurrent prostate cancer prolonged the time to disease progression but did not eradicate the disease [124].

On the other hand, pTVG-AR, encoding the ligand-binding domain of androgen receptors, targets the androgen receptor (AR), which plays a pivotal role in the development and progression of prostate cancer, particularly in castration-resistant prostate cancer (CRPC). By inducing an immune response against AR-expressing cells, pTVG-AR with GM-CSF aims to combat tumor growth even in the context of hormone therapy resistance. A multicenter phase I trial indicated that this vaccine could successfully stimulate an AR-specific Th1 immune response and prolong PSA progression-free survival (PFS) by 18 months[125].

One of the two experimental arms of a randomized, open-label, multicenter study described the administration of two DNA vaccines, pTVG-HP and pTVG-AR, combined with pembrolizumab. This trial evaluated PFS as a primary outcome with several secondary outcomes. The completion date is estimated to be December 2025.

#### Breast cancer

WOKVAC is a DNA plasmid-based vaccine targeting three proteins—IGFBP2, HER2, and IGF-1R—that are linked to the progression of high-risk breast lesions to invasive breast cancer. A phase I trial (NCT02780401) evaluating the side effects and optimal dosing of pUMVC3-IGFBP2-HER2-IGF1R combined with Sargramostim in patients with nonmetastatic, node-positive, HER2-negative breast cancer aimed at preventing cancer recurrence [126]. Additionally, a phase II single-arm trial (NCT04329065) investigated the efficacy of WOKVAC combined with neoadjuvant taxane-based chemotherapy and HER2 antibody therapy (either TCHP or THP) in stage I-III HER2 + (HR ±) breast cancer patients. This study included 16 patients who were administered WOKVAC on day 13 of cycles 1–3 to assess whether it enhances T-bet + CD4^+^ and CD8^+^ TILs and examined the safety and immune response posttreatment, focusing on its association with pathological responses [127].

The DNA plasmid-based therapeutic vaccine STEMVAC is currently being tested in multiple clinical trials targeting different cancer types, including breast cancer and non-small cell lung cancer (NSCLC). A phase I trial (NCT02157051) focusing on HER2-negative advanced-stage breast cancer patients is underway to assess the safety and immunogenicity of STEMVAC. This trial began in June 2015 and aimed to enroll approximately 30 women with stage III or IV breast cancer [128]. Additionally, there is an ongoing phase II trial (NCT05242965) evaluating the efficacy of STEMVAC in shrinking tumors in patients with stage IV NSCLC. This trial tested a vaccine in combination with GM-CSF, which is used as an adjuvant to enhance the immune response. The aim is to activate immune cells that recognize and destroy lung cancer cells. Another ongoing phase II trial (NCT05455658) explored the immunogenicity of STEMVAC in patients with early-stage triple-negative breast cancer. This study investigated whether STEMVAC can activate the immune system in patients previously diagnosed with and treated for triple-negative breast cancer.

#### Cervical cancer

Currently, several clinical trials are investigating the efficacy and safety of DNA vaccines encoding HPV E6 and E7 for the treatment of cervical cancer. Early-phase trials have demonstrated the potential of these vaccines to elicit strong CTL responses against HPV-infected cells [129]. For example, VGX-3100 is a therapeutic DNA vaccine designed to treat high-grade squamous intraepithelial lesions (HSILs) caused by HPV types 16 and 18, which are often precursors to cervical cancer. VGX-3100 has been reported to induce regression of cervical intraepithelial neoplasia (CIN) lesions and clearance of HPV-16/18 infection in a significant proportion of treated individuals in phase II trials (NCT01304524) [130].

In the REVEAL 1 phase 3 trial, compared with placebo, VGX-3100 significantly ameliorated HSIL and eradicated HPV-16 and/or HPV-18 infections in a significant proportion of patients. This trial also highlighted the safety and tolerability of the vaccine, with most adverse events being mild to moderate and primarily related to injection site reactions [131]. In addition, REVEAL 2 was fully enrolled, and top-line efficacy and safety data are expected to be available soon. These trials are critical for potentially establishing VGX-3100 as a nonsurgical therapeutic option for cervical HSIL, a need highlighted by the absence of nonsurgical treatments for this condition [132].

In recent phase II clinical studies (NCT03444376), another DNA vaccine, GX-188E, encoding HPV E6/E7, combined with pembrolizumab, showed promising results in treating HPV 16- and 18-positive advanced cervical cancer. The studies reported an overall response rate of 31.3%, with a complete response rate of 10.4% and a partial response rate of 20.8%. The median overall survival was 16.7 months, and the regimen was safe and tolerable [133]. A phase 3 clinical trial is recommended further to explore the efficacy and safety of this combination.

#### Personalized neoantigens

Although TAAs are present in both normal and germline tissues, they often lead to immune tolerance, which can hinder robust immune activation. This has resulted in several clinical trials using nonmutated tumor antigens that have failed to show significant benefits over standard care treatments [134]. In contrast, neoantigens arise from unique tumor-specific DNA mutations that create novel epitopes exclusively expressed in cancer cells. This specificity minimizes potential side effects and makes neoantigens ideal for developing targeted cancer vaccines. Importantly, since neoantigens are perceived as nonself by the immune system, they bypass the issue of T-cell tolerance, fostering a robust antitumor response [135]. In recent years, personalized neoantigens have been evaluated in various cancer DNA vaccine studies. During this process, mutations are analyzed by exome and RNA sequencing of samples collected from tumor tissue or PBMCs. Antigenic epitopes were determined by bioinformatics, MHC-peptide binding assays, and ELISOPT methods [135]. Based on these findings, plasmid DNA vaccines that encode multiple neoantigens have been constructed and administered to individual patients [136].

Previous preclinical research has explored polyepitope neoantigen DNA vaccines, which aim to trigger a comprehensive immune response [137]. These studies have shown promising results, including increased production of interferon-gamma (IFN-γ), enhanced helper T-cell (Th) and cytotoxic T-lymphocyte (CTL) responses, and notably, a reduction in tumor growth and metastasis across various cancer models [138]. Additionally, many clinical trials are testing the safety and efficacy of neoantigen DNA vaccines, as listed in Table 2.

In particular, in clinical studies NCT02348320 and NCT03199040, researchers explored personalized polyepitope vaccines aimed at treating breast cancer, while study NCT03122106 applies the same approach to treating pancreatic cancer. These studies are important because they are testing a new strategy that could address two significant challenges in cancer treatment: tumor heterogeneity (the variation within tumors) and the loss of immunogenicity in TAAs. The loss of immunogenicity in TAAs is a key reason many cancer treatments fail [139, 140]. By tailoring vaccines to the specific characteristics of each patient's tumor, this approach could enhance the effectiveness of cancer immunotherapy.

## Advances in mRNA-based cancer vaccines

### Development of mRNA vaccines

The development of mRNA vaccines has made revolutionary strides in the fields of immunology and biomedical therapeutics. Researchers successfully demonstrated the expression of various proteins in mice through direct injection of mRNA into muscle tissue in the early 1990s [141]. This foundational work led to the first mRNA-based vaccine trials in 1993, which generated an anti-influenza CTL response in mice [142]. By 1995, mRNA vaccines encoding cancer antigens have been tested, showing the ability of mRNAs to induce in situ protein expression and specific immune responses [143, 144]. Despite these early successes, the development of mRNA vaccines has encountered significant hurdles, primarily due to the inherent instability and susceptibility of these molecules to rapid degradation. For nearly a decade, progress was sluggish, as the scientific community turned its focus toward more stable DNA-based alternatives. It was not until advances in cell-free synthetic processes, including IVT from DNA templates, that the field regained momentum. These processes allowed the generation of highly pure and stable mRNA ready for therapeutic use.

In recent years, innovations such as the incorporation of modified nucleosides and the development of robust delivery mechanisms such as LNPs have addressed critical challenges of stability and immunogenicity. These advancements have significantly enhanced the clinical applicability of mRNA vaccines, culminating in their successful deployment against COVID-19. This breakthrough has not only validated the potential of mRNA vaccines in pandemic response but has also catalyzed further research into their use against a broad spectrum of diseases, including infectious diseases such as flu [145, 146], Zika virus [147, 148], and other viruses [149], as well as potential applications in treating autoimmune diseases [150] and allergies [151]. The future of mRNA vaccines is promising, yet challenges remain, particularly in optimizing their formulation to allow for easier distribution and storage, reducing the dependence on ultracold supply chains, and ensuring broader global access. Continued research is essential to refine mRNA vaccine platforms to enhance their stability, minimize potential side effects, and expand their therapeutic reach, potentially transforming the landscape of disease prevention and treatment.

### Strategies for mRNA vaccine optimization

**Fig. 3:**
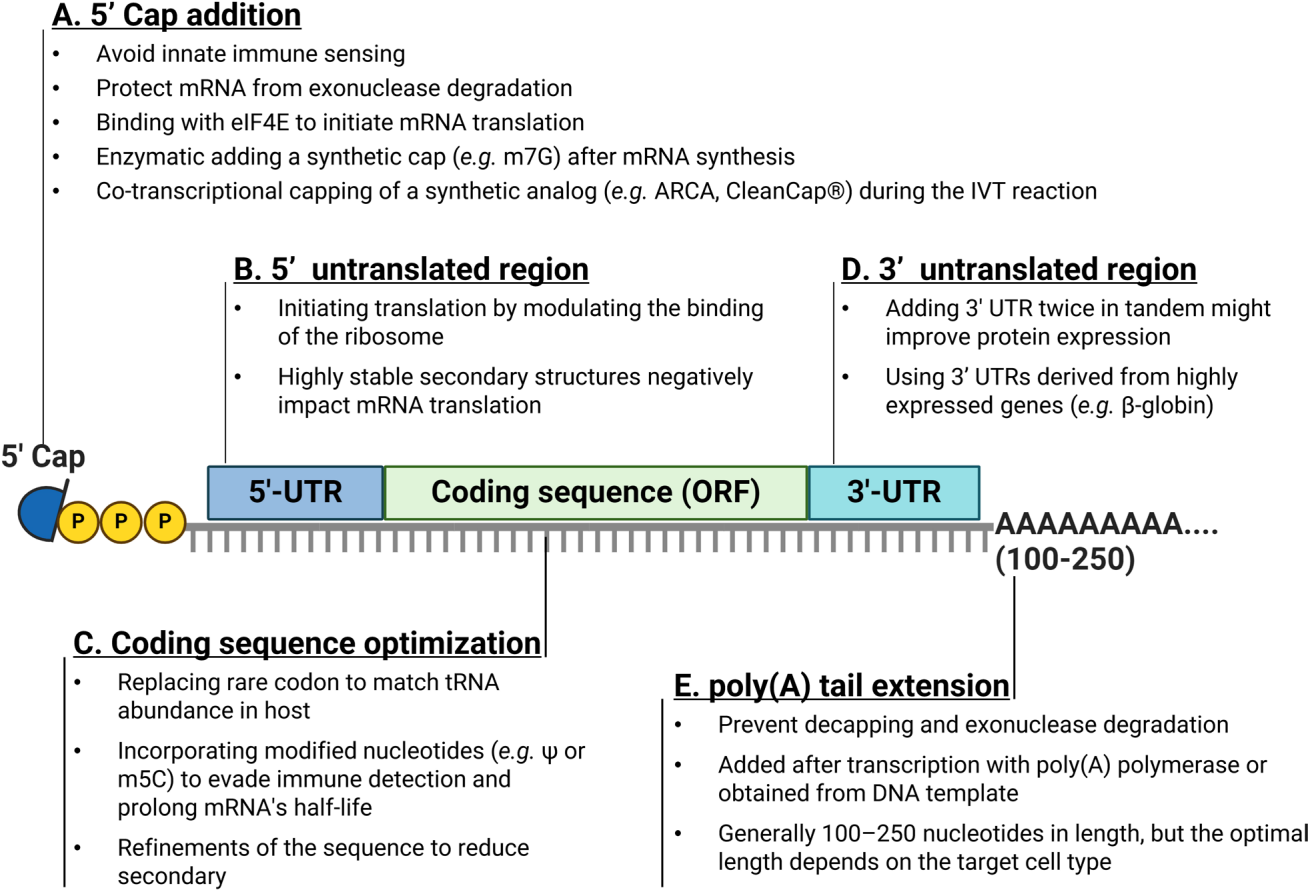
Essential Components and Strategies for mRNA Vaccine Design. Illustration of the key structural and strategic elements in the design of an mRNA vaccine to optimize its stability and efficacy. The components include **A** 5' cap addition: aids in evading innate immune detection, protects mRNA from exonuclease degradation, and facilitates translation initiation. **B** 5' Untranslated Region (UTR): This region modulates ribosome binding to initiate translation, but stable secondary structures can negatively influence mRNA translation. **C** Coding sequence optimization involves replacing rare codons to match tRNA abundance in the host, using modified nucleotides to avoid immune detection and extend the mRNA half-life, and refining the sequence to reduce secondary structure. **D** 3' Untranslated Region (UTR): 3' UTR sequences were added in tandem to improve protein expression, and UTRs from highly expressed genes were used to increase overall mRNA effectiveness. **E** Poly(A) tail extension: poly(A) tails are added posttranscriptionally or encoded directly within the DNA template, with lengths generally between 100–250 nucleotides tailored to the target cell type to prevent mRNA decapping and exonuclease degradation. (Figure created with Biorender)

Since mRNA is inherently unstable, it can be quickly degraded enzymatically and requires careful handling and formulation to ensure effective delivery and expression within host cells. To improve mRNA/LNP vaccine efficacy, some new designs have been developed that incorporate mRNA molecules and LNP components (Fig. 3).

#### Modification of the mRNA structure

The key structural elements of mRNA that can be optimized to maintain the activity of mRNA therapeutics include the cap structure, untranslated regions (UTRs), and polyadenylated (poly(A)) tail. The cap structure of mRNA is critical for the recognition of the cellular translation machinery, interaction with eukaryotic translation initiation factor 4E (eIF4E), and utilization of the internal ribosome entry site (IRES) for alternative translation initiation [152]. The 5′ m7G cap is an evolutionarily conserved modification of eukaryotic mRNA. Two methods could be applied to generate capped mRNA in vitro. One uses transcript RNA as a template followed by three sequential enzymatic steps to modify the N7 amine of the guanosine cap by guanine-N7 methyltransferase activity [153]. The other employs modified cap analogs such as the anti-reverse cap analog (ARCA) and CleanCap Reagent AG (5´-m7G joined by a 5´-5´ triphosphate linkage to an AG sequence) during in vitro transcription. It is a simple process with a high recovery rate of mRNA. The stability and translational efficiency of the mRNA are significantly improved. Moderna used three sequential enzyme methods to produce a COVID-19 mRNA vaccine. Cotranscription capping methods were used by BioNtech/Pfizer to generate COVID-19 mRNA vaccines [154].

In addition to 5’-end capping, the substitution of natural nucleosides with modified counterparts such as pseudouridine (Ψ) and 1-methyl pseudouridine (m1Ψ) in the mRNA sequence plays a critical role in reducing its recognition by innate immune sensors such as TLR7. The discovery that modified nucleosides in mRNAs can reduce innate receptor activation and increase protein expression is one of the key innovations in mRNA vaccines, which was discovered by Katalin Karikó and Drew Weissman [155]. The breakthrough findings by the two 2023 Nobel Laureates are critical for developing effective mRNA vaccines against COVID-19 [156]. These modifications not only diminish the immunogenicity of mRNA but also improve its translational profile, contributing to more sustained expression of the encoded protein. Moreover, the 5’-UTR and 3’-UTR are optimized to enhance mRNA stability by incorporating sequences from highly stable genes or by adding binding sites for stabilizing proteins [157]. These optimizations help protect the mRNA from rapid degradation by cellular nucleases. Moreover, the design of the poly(A) tail is also crucial. Modifications to its length and composition can significantly impact mRNA stability and efficiency, with longer tails generally associated with enhanced translational capacity and increased half-life of the mRNA molecule [158]. The interplay between poly(A) tails and poly(A)-binding proteins is critical for protein translation and mRNA decay [159]. Recent global analyses have demonstrated that the poly(A) tail of many mRNAs may actually contain approximately 50–100 nucleotides [160]. Most scientists believe that the optimal poly(A) tail length for mRNA synthesis in vitro is 100–150 nucleotides.

Collectively, these strategic modifications to the mRNA structure ensure that the vaccines not only remain stable and less prone to degradation but also maintain high efficiency in protein translation, which is crucial for eliciting a robust and effective immune response. This tailored approach to mRNA vaccine design is fundamental for harnessing their full potential for preventing diseases and treating various medical conditions, including cancer.

#### Optimization of the coding sequence

Optimizing the coding sequence of mRNA vaccines is a critical strategy for enhancing their stability and translational efficiency, thereby improving their overall therapeutic efficacy. Codon optimization involves tailoring the mRNA sequence to match the codon usage preferences of the host’s cellular machinery. By selecting codons that are frequently used in highly expressed genes of the host, the mRNA is more efficiently translated, contributing to increased stability and higher protein yield [11].

In addition to codon optimization for the host, another approach to codon optimization involves adjusting the guanine (G) and cytosine (C) contents within the open reading frame (ORF). This modification aims to regulate the rate of translation elongation, as a balanced GC content can enhance mRNA structural stability and protect against endoribonuclease degradation [161]. Moreover, reducing uridine-rich sequences can mitigate recognition and activation by RIG-I-like receptors, which can degrade mRNA and halt protein synthesis [162]. Instead, optimizing the sequence to mirror the codon proportion found in abundantly expressed host proteins or selecting the best pairs of codons known to be effective in these contexts can be advantageous [163, 164].

Furthermore, replacing rare codons with those corresponding to more abundant tRNAs can significantly increase the translation rate, ensuring that the protein synthesis machinery operates more smoothly and effectively. However, care must be taken to avoid the formation of stable secondary structures, such as hairpin loops within the mRNA, which can impede translation [165].

Although codon optimization can markedly improve mRNA vaccine performance by enhancing protein expression and stability, it also has challenges. Changes in codon usage can alter mRNA secondary structure, affect translational kinetics, and potentially influence protein folding [166, 167]. These alterations may impact the immunogenicity of the encoded antigen, underscoring the need for careful design and thorough evaluation of optimized mRNA vaccines to ensure that they maintain the desired safety and efficacy profiles without unintended biological effects.

#### Delivery of stable formulations

Employing stable formulations that ensure efficient intracellular delivery can significantly enhance the stability and efficacy of mRNA-based vaccines. Cationic lipid-composed liposomes, lipoplexes, cationic polymers, and ionizable lipid-composed LNPs are carriers applied preclinically and in clinical trials for mRNA delivery [168]. These lipid-based carriers in the formulation of mRNA vaccines provide a robust barrier to protect mRNA molecules from enzymatic degradation and facilitate their delivery into the cytosol [169]. This protective mechanism is crucial, considering the inherently unstable nature of mRNA and its susceptibility to rapid degradation by exonucleases and endonucleases [170].

#### Liposome

Cationic liposomes comprise positively charged lipids, facilitating the encapsulation and protection of negatively charged mRNA molecules. DOTMA (1,2-di-O-octadecenyl—3-trimethylammonium propane) and DOTAP (1,2-dioleoyl—3-trimethylammonium-propane) are the two most widely used cationic lipids [171]. These lipids remain positively charged at all physiological pH values and can easily condense anionic mRNA [172]. They also promote strong electrostatic interactions with cell membranes, enhancing cellular uptake through endocytosis and leading to effective delivery and robust protein expression. In addition to their delivery efficiency, DOTMA and DOTAP exhibit intrinsic adjuvant properties, amplifying the immune response to mRNA and making them highly suitable for vaccine applications [173]. The clinical efficacy of DOTAP-based liposomes has been demonstrated in various trials [174], including the successful delivery of COVID-19 mRNA vaccines [175].

However, the use of DOTMA or DOTAP is accompanied by particular challenges. Serum instability and limited long-term stability necessitate cold chain storage [171, 176], which poses logistical challenges. A high positive charge density can induce cytotoxicity and inflammatory responses [172, 177], which require careful management in clinical settings.

#### Polymers

Polymers, particularly those displaying a high positive charge density associated with amino groups, enhance mRNA vaccine stability by condensing and encapsulating negatively charged mRNA through electrostatic interactions under acidic conditions. These polymers can be easily functionalized with targeting peptides and responsive materials, allowing for targeted delivery and controlled release of mRNA [178]. This adaptability makes polymers an effective alternative to lipid-based systems for protecting mRNA vaccines from degradation and improving delivery efficiency.

Despite their potential, polymer-based delivery systems are less clinically advanced than lipid-based systems, partly due to polydispersity and metabolic challenges [179]. Polyethyleneimine (PEI) is a common cationic polymer for mRNA transfection but poses toxicity issues due to its high molecular weight. High-molecular-weight polymers, such as PEI and chitosan, often require modifications to improve transfection efficacy and durability, influenced by factors such as polymer length, charge density, and concentration [180]. Efforts to mitigate these issues include conjugating PEI with cyclodextrin, which reduces cytotoxicity while maintaining transfection efficiency [181].

Additionally, researchers have found that biodegradable polymers such as poly(lactic-co-glycolic acid) (PLGA) offer low toxicity and good stability but have low mRNA encapsulation efficiency due to their anionic nature at physiological pH [182]. Hyperbranched dendrimers with high amine density efficiently form mRNA complexes, although modifications with polyethylene glycol (PEG) or disulfide linkages mitigate toxicity issues [183]. pH-responsive polymers release mRNA upon cytoplasmic entry by self-degradation, while amphipathic polymers form nanocomplexes by electrostatic binding to mRNA [184].

#### Lipid nanoparticles

Lipid nanoparticles (LNPs) have emerged as a cornerstone in the formulation of mRNA vaccines due to their exceptional ability to encapsulate mRNA within a lipid-based structure, providing a robust barrier against ribonucleases and facilitating their delivery into the cytosol. LNPs generally comprise four components: an ionizable lipid that dictates particle charge, cholesterol, a helper lipid, and PEGylated lipids that aid in particle stability, formation, and membrane–membrane fusion in the cell. The lipid composition of LNPs allows them to merge seamlessly with cellular membranes. This integration enables the direct translocation of mRNA into the cytoplasm, bypassing the endosomal pathway and reducing the risk of degradation within lysosomal compartments. Once inside the cell, the encapsulated mRNA is released, allowing translation of the encoded antigens and subsequent induction of a targeted immune response [185].

Furthermore, the versatility of LNPs allows for their optimization in terms of size, charge, and surface properties. Particle size is directly linked to biodistribution. Particle sizes between 200 and 400 nm tend to accumulate in the spleen after intravenous administration, whereas smaller particles, ideally between 20 and 50 nm, are necessary for effective trafficking into lymph nodes [186, 187]. By adjusting these parameters or incorporating specific targeting ligands, LNPs can be tailored to enhance cellular uptake and direct delivery to specific cell types or tissues [188, 189]. This targeted delivery not only improves the efficacy of the vaccine but also minimizes potential off-target effects [190], making LNPs an indispensable tool for developing advanced mRNA vaccine formulations. These strategies collectively enhance mRNA vaccine stability, delivery efficiency, and therapeutic potential, which are critical for their application in preventing infectious diseases and treating conditions such as cancer.

### Clinical studies of mRNA cancer vaccines

The first therapeutic cancer vaccine using mRNA transfection into DCs marked the beginning of mRNA-based cancer vaccines in clinical trials [191]. Although these DC-based mRNA vaccines still dominate clinical research, there has been a significant shift toward exploring intravenously administered mRNA-based immunotherapies using nonviral vectors. This shift is driven by encouraging antitumor results from preclinical studies, with companies such as CureVac, BioNTech, and Moderna leading the efforts. Numerous mRNA-based cancer vaccines that target either personalized neoantigens or a combination of TAAs are currently under clinical evaluation (Table 3). These vaccines are being tested against a range of challenging cancers, such as non-small cell lung cancer (NSCLC), colorectal carcinoma (CRC), and melanoma, which are known for their aggressive and often metastatic nature.

**Table 3:** mRNA vaccines for cancer immunotherapy in clinical trial

Collaborator/Sponsor	Brand	Antigen	Combination therapy	Cancer type	Start	Phase	NCT	Status
CureVac	CV9201	TAAs: MAGEC1, MAGEC2, NY-ESO-1, survivin, 5T4		NSCLC	2009	I/II	NCT00923312	Completed
	BI1361849 (CV9202)	TAAs: NY-ESO-1, MAGE-C1, MAGE-C2, 5T4, survivin, and MUC1	local radiation	NSCLC	2013	I	NCT01915524	Terminated
			Durvalumab Tremelimumab	NSCLC	2017	I/II	NCT03164772	Completed
	CV9103	TAAs: PSA, PSCA, PSMA, and STEAP1		prostate carcinoma	2009	I/II	NCT00831467	Completed
	CV9104	TAAs: PSA, PSCA, PSMA, STEAP1, PAP and MUC1		prostate carcinoma	2014	II	NCT02140138	Terminated
Moderna	mRNA-2416	Human OX40L	Durvalumab (a-PD-L1 Ab)	solid tumor maliganancies or lymphoma	2017	I/II	NCT03323398	Terminated
	mRNA-2752	Human OX40L/IL-23/IL-36γ	Durvalumab	Solid Tumor Malignancies or Lymphoma	2018	I	NCT03739931	Active, not recruiting
	mRNA-4157	Personalized neoantigen	Pembrolizumab (a-PD-1 Ab)	Solid tumor	2017	I	NCT03313778	Recruiting
			Pembrolizumab	Cutaneous melanoma	2019	II	NCT03897881	Recruiting
National Cancer Institute (NCI)	mRNA-4650	Personalized neoantigen		Melanoma, Colon cancer Gastrointestinal cancer Genitourinary cancer Hepatocellular cancer	2018	I/II	NCT03480152	Terminated
Merck Sharp & Dohme	mRNA-5671 /V941	KRAS mutations: G12D, G12V, G13D, G12C	V941 Pembrolizumab	NSCLC, Pancreatic Neoplasms Colorectal Neoplasm	2019	I	NCT03948763	Completed
	mRNA-4157 /V940	Personalized neoantigen	Pembrolizumab	Melanoma	2023	III	NCT05933577	Recruiting
BioNTech	IVAC MUTANOME	Individualized poly-neo-epitope	RBL001/RBL002	Melanoma	2013	I	NCT02035956	Completed
	W ova1	TAA		Ovarian cancer	2019	I	NCT04163094	Terminated
	BNT-111	4 TAAs: NY-ESO-1, tyrosinase, MAGE-A3, and TPTE	Lipo-MERIT	Advanced melanoma	2021	I	NCT02410733	Completed
			Cemiplimab (a-PD-1 Ab)	Unresectable melanoma	2021	II	NCT04526899	Active, not recruiting
	BNT-112	5 TAAs: kallikrein-2, kallikrein-3, acid phosphatase prostate, homeobox B13 (HOXB13), and NK3 homeobox 1	Cemiplimab	Prostate cancer	2019	I/II	NCT04382898	Active, not recruiting
	BNT-113	HPV16( +) E6/E7	Pembrolizumab	Head and Neck Cancer	2021	II	NCT04534205	Recruiting
	BNT-116	TAA	Cemiplimab Docetaxel Carboplatin Paclitaxel	NSCLC	2022	I	NCT05142189	Recruiting
	BNT-122	RO7198457	/	Colorectal cancer	2021	II	NCT04486378	Recruiting
BioNTech/ Genentech	RO7198457	Individualized neoantigen	Atezolizumab (a-PD-L1 Ab)	Melanoma NSCLC Bladder cancer	2017	I	NCT03289962	Active, not recruiting
			Atezolizumab	NSCLC	2021	II	NCT04267237	Withdrawn
			Pembrolizumab	Advanced Melanoma	2019	II	NCT03815058	Active, not recruiting
Stemirna Therapeutics	SW1115C3	Personalized neoantigen		Solid tumor	2022	I	NCT05198752	Recruiting
Gritstone bio	**GRANITE** GRT-C901 GRT-R902	Patient-specific neoantigens	Nivolumab Ipilimumab (a-CTLA-4 Ab)	NSCL Cancer Colorectal Cancer Gastroesophageal Adenocarcinoma Urothelial Carcinoma	2019	I/II	NCT03639714	Completed
			Atezolizumab Ipilimumab Fluoropyrimidine plus leucovorin Bevacizumab	Colorectal Neoplasms	2022	II/III	NCT05141721	Active, not recruiting
			Atezolizumab Ipilimumab	Colonic Neoplasms Colorectal Neoplasms	2022	II	NCT05456165	Terminated
	**SLATE** GRT-C903 GRT-R904	20 shared neoantigens from KRAS, TP53, β-catenin, and BRAF genes	Nivolumab Ipilimumab	NSCL Cancer Colorectal cancer Pancreatic cancer Solid tumor Shared Neoantigen-Positive Solid Tumors	2019	I/II	NCT03953235	Completed

#### CureVac

CV9201, an RNActive®-based cancer immunotherapy, was tested in a phase I/IIa trial involving 46 patients with advanced NSCLC. Naked mRNA vaccines encoding five specific lung cancer antigens were intradermally co-delivered with protamine/mRNA complexes, which are known to have self-adjuvant properties. The results indicated that the therapy was well tolerated at all doses, with the most common side effects being mild injection site reactions and flu-like symptoms. Notably, 63% of patients develop antigen-specific immune responses, and this therapy has potential for further clinical development [192].

This new collaboration focused on CureVac’s CV9202 in the study of combining active and passive immunotherapies for NSCLC. BI 1361849, a cancer vaccine with six mRNA components targeting NSCLC antigens, was tested alongside durvalumab, a checkpoint inhibitor that blocks PD-L1. This trial also evaluated the combination of durvalumab with tremelimumab, an anti-CTLA-4 antibody, potentially enhancing immune response effectiveness. This phase 1/2 trial (NCT03164772) assessed the safety, efficacy, and optimal dosages of these therapies, with patients divided into two treatment arms and a control group. Treatments span 12 cycles, with checkpoint inhibitors administered intravenously and the vaccine given intradermally. Primary outcomes focus on safety and tolerability, while secondary outcomes measure treatment efficacy through various clinical parameters[193].

#### Moderna

Another pioneering player, Moderna, is advancing its innovative mRNA technology with two products designed for cancer treatment and is currently undergoing phase I clinical trials. The first product, mRNA-2416, encodes OX40L and is being tested either alone or combined with the intravenously administered PD-L1 inhibitor durvalumab to treat lymphoma and metastatic ovarian cancer. Early findings from these trials indicate that mRNA-2416 is tolerable at various dosages without severe adverse effects, showing potential for further investigation of combination therapies with durvalumab in solid tumors [194]. The second product, mRNA-2752, combines mRNAs for OX40L, IL-23, and IL-36γ, aiming to enhance T-cell functionality and promote proinflammatory responses to combat cancer more effectively. This product is also under study for its use in lymphoma and shows promise in initial results when used with durvalumab. It is well tolerated and exhibits signs of antitumor activity [195].

In collaboration with Merck, Moderna is conducting a phase I trial of mRNA-5671 (NCT03948763), a personalized vaccine targeting KRAS neoantigens [196]. This vaccine is delivered intramuscularly every three weeks for nine cycles, and LNPs are used for efficient delivery. It is being tested alone and in combination with Merck's PD-1-specific antibody, pembrolizumab, for treating pancreatic cancer. The preliminary results indicate that the vaccine has been well tolerated and has stimulated an antitumoural immune response.

Another Moderna product, mRNA-4157, is being tested as a personalized vaccine for patients with resected solid tumors, including melanoma, bladder carcinoma, and non-small cell lung cancer (NSCLC) [197]. This vaccine is being tested both as a monotherapy and in combination with pembrolizumab (NCT03313778). Early results from these trials showed that mRNA-4157 has an acceptable safety profile and induces significant neoantigen-specific T-cell responses. Notably, twelve of thirteen patients treated with the monotherapy regimen remained disease-free [198]. Recent findings from the Phase IIb KEYNOTE-942/mRNA-4157-P201 trial (NCT03897881) demonstrated the effectiveness of mRNA-4157 (V940) vaccine in combination with pembrolizumab for treating high-risk stage IIIB/C/D and IV melanoma. The combination therapy showed a 44% risk reduction in recurrence or death compared to pembrolizumab alone. Serious treatment-related adverse events were reported in 14.4% of patients receiving the combination [199, 200]. The trial’s success led to a breakthrough therapy designation by the FDA and EMA. A follow-up Phase III trial, V940-001 (NCT05933577), is planned to assess the efficacy in high-risk melanoma patients further.

#### BioNtech

BNT111, developed by BioNTech, is a therapeutic mRNA vaccine designed to treat patients with advanced melanoma. Leveraging the technology from the Lipo-MERIT platform involves complexing mRNA with cationic lipids. BNT111 targets four melanoma-associated antigens known for their high prevalence and immunogenicity in melanoma [201]. The vaccine has shown promise in early clinical trials, including a phase I dose-escalation trial (NCT02410733), demonstrating that a significant proportion of participants exhibited an immune response. This trial also indicated the potential efficacy of BNT111 in inducing both CD4- and CD8-positive T-cell responses, which is crucial for combating melanoma [202]. Currently, BNT111 is being further tested in a randomized phase II clinical trial (NCT04526899) to assess its effectiveness as a monotherapy and in combination with cemiplimab, an anti-PD-1 antibody, especially for stage III and IV melanoma that is refractory or has relapsed after standard treatments. This ongoing research underscores the role of this vaccine as one of the most promising cancer immunotherapies under development for melanoma treatment.

A phase I/II clinical trial (NCT04382898) is evaluating the cancer vaccine BNT112, which targets TAAs, for treating metastatic prostate cancer. This trial explored the effectiveness of the vaccine alone and in combination with the drug cemiplimab. Currently, an innovative mRNA-based vaccine platform called iNeST (individualized neoantigen-specific immunotherapy), also known as BNT122, is under investigation. This platform designs vaccines customized for each patient's tumor mutations. For instance, one trial is assessing iNeST alongside another RNA-based therapy for patients with triple-negative breast cancer, exploring new avenues in cancer treatment. Additionally, BioNtech collaborated with Genentech to join the campaign and evaluate the safety and efficacy of the mRNA personalized vaccine RO7198457 delivered by the Lipo-MERIT platform in multiple phase I and II clinical trials [203].

## Challenges, opportunity, and future directions

Numerous past and ongoing clinical studies are exploring the potential of DNA and mRNA cancer vaccines using various strategies. These studies involve the incorporation of TAAs, tumor-specific antigens (TSAs), or neoantigens, often in combination with cytokines, immune checkpoint blockade (ICB), chemotherapy, and targeted therapies. Additionally, combinations with drugs that inhibit the tumor microenvironment are under investigation. Despite the promising potential of DNA and mRNA cancer vaccines, several challenges still need to be addressed.

### DNA delivery

DNA vaccines delivered by electroporation have been shown to induce high levels of cellular and humoral immunity in animals. However, an extra medical device is necessary for DAN delivery. The design of medical devices for human use is complicated and needs to follow the regulatory guidelines for medical devices. Some electroporation machines have been used in humans, including the CELLECTRA® device (Inovio Biopharmaeuticals, Inc.), TriGrid™ System (Ichor Medical Systems), and DERMA VAX™ device (Cyto Pulse Sciences). Previously, we applied an electroacupuncture machine with a disposable needle, which has been approved for phase I clinical trials. These electrical devices can induce strong cellular and humoral immune responses [68]. However, electric devices are not convenient for clinical applications, and developing a nonelectric DNA delivery system would be a future research direction. Recently, several reports have demonstrated that DNA vaccines formulated with ionizable lipid nanoparticles (DNA/LNPs) can induce protective immunity against SARS-CoV-2 infection [81–83]. These results suggest that the DNA delivered by using ionizable LNPs could be applied to future vaccine development. However, DNA/LNP formulations must be optimized to improve efficiency. A unique barcoded DNA system has been used to optimize LNP formulations for DNA delivery [204], and a better formulation has been reported [82]. Although plasmid DNA delivered via LNPs (pDNA-LNPs) commonly induces inflammation driven by activation of the cGAS-STING signaling pathway, co-loading endogenous lipids that inhibit STING into pDNA-LNPs mitigated serious inflammatory responses in vivo, enabling prolonged transgene expression [205]. It can be expected that new LNP formulations for DNA cancer vaccines will be intensively studied in the future.

### mRNA stability

Enhancing the stability of mRNA/LNP formulations both in vitro and in vivo remains a significant challenge in the development of cancer vaccines. Typically, mRNA/LNP storage requires ultralow temperatures, such as -20 °C or -70 °C, and this requirement for cold chain transportation significantly hinders the widespread distribution of mRNA/LNP-based vaccines. Currently, long-term storage of these vaccine samples at 4 °C is impractical. However, some interesting studies have shown that smaller mRNA/LNP particles (60–80 nm) exhibit higher stability at 4 °C compared to their larger counterparts (120–150 nm) [206]. Recent advancements include the development of novel ionizable cationic lipids that could potentially improve mRNA/LNP stability. Additionally, optimal concentrations of sucrose and sodium phosphate have proven effective in stabilizing mRNA/LNPs in current COVID-19 vaccines and in preclinical studies of self-amplifying mRNA vaccines [154, 207].

In addition to its stability during storage, The inherent instability of mRNA in vivo presents another significant challenge. It is known that mRNA is very unstable in circulation due to its chemical and physical properties. Chemically, mRNA is susceptible to degradation by RNase, ribozymes, and acids via similar pathways. Physically, the propensity of mRNA to aggregate or precipitate due to its secondary or tertiary structures further complicates its stability, resulting in reduced translational efficiency. [208]. Although the current mRNA vaccines have been modified through 5’-capping, 5’-and 3’-UTR, modified uridine, and poly-A tail to increase mRNA stability, other approaches have been used to increase the stability of mRNA vaccines [209]. It has been reported that the length of an mRNA is negatively correlated with its half-life [210]; high GC content plays a central role in mRNA translation and stability, as degradation of high GC content mRNA is controlled by the inefficient helicase DDX6 and 5' − 3' exonuclease XRN1A. [211]. Additionally, optimizing the codons used in mRNA sequences can further improve stability and protein translation efficiency [212]. In recent research, circular RNA (circRNA) has received much attention because circRNA is covalently closed by reverse splicing at the 3′ and 5′ ends; this structural attribute makes circRNA much more stable than linear mRNA [213]. However, the production of circRNA is currently limited by complex manufacturing processes and low yields. Addressing these manufacturing challenges is essential for the future use of circRNA in cancer immunotherapy.

### Manufacturing of clinically used DNA/mRNA

Personal DNA/mRNA cancer vaccines represent a promising frontier in oncology, leveraging the power of sequencing and computational predictions to tailor treatments to individual patients. However, their production poses significant challenges, particularly regarding compliance with Good Manufacturing Practices (GMP) and cost-efficiency. The synthesis of GMP-grade DNA/mRNA is a labor-intensive and expensive process. This is compounded by the relatively small amount of material required per patient, rendering traditional large-scale GMP manufacturing approaches inefficient.

To address these production challenges, innovative strategies involving scaled-down, automated processes are being explored. One potential approach is the development of compact, automated production systems that integrate small-volume processing with continuous online monitoring. Such systems would streamline various stages of vaccine production, including E. coli fermentation, DNA purification, DNA linearization, IVT of mRNA, and mRNA purification. Following these steps, the purified mRNA could be processed in a separate facility for LNP formulation and filling. The objective is to streamline the vaccine production, which can be available within one month of manufacturing initiation. Moreover, the target cost for these personalized vaccines should ideally not exceed USD 50,000 per patient, making them a viable option for broader use.

Moderna's introduction of the "single-use personal RNA + " machine is a notable advancement in this field. This compact, refrigerator-sized unit can encode up to 34 potential neoantigens for each mRNA, specifically targeting individual tumors [214]. This technology represents a significant shift towards more personalized and rapid production of cancer vaccines, potentially transforming future cancer treatment paradigms.

### Antigen selection

TAAs typically exhibit low immunogenicity, posing a challenge in eliciting robust antitumor responses. This is primarily because TAA-specific T cells are often eliminated during T-cell maturation or develop peripheral tolerance. Historically, many DNA and mRNA cancer vaccines have targeted TAAs, but these have shown limited therapeutic efficacy. In contrast, recent strategies have shifted towards employing TSAs with higher immunogenicity. These include viral antigens and neoantigens, which are currently being explored in various clinical trials [215]. A notable example is the HPV E6/7 DNA vaccine (INO-3107), employed in treating recurrent respiratory papillomatosis and has become a breakthrough therapy in the United States. [216]. Additionally, the evaluation of VGX-3100 alongside electroporation for treating cervical HSIL (NCT03185013) was initiated in 2017 by Inovio Pharmaceuticals [217]. Neoantigens, which can provoke strong immune responses in individual patients, represent a promising avenue. However, their identification is costly and typically specific to each patient, which limits broader applicability. A critical issue is determining the optimal number of neoantigens to include in vaccines and identifying those with the highest antigenicity. To overcome these challenges, leveraging big data to identify common or shared neoantigens across different cancers or patients might provide a more cost-effective approach. These shared neoantigens could be pooled for use in broader patient populations. Current methodologies, such as next-generation sequencing, mass spectrometry, and AI-assisted analysis, facilitate the identification of potential neoantigens. Nevertheless, a significant limitation remains the inadequate immune response elicited by many predicted neoantigens, underscoring the need for designing simple yet effective assays to validate their immunogenicity. Furthermore, proteins exhibiting high mutation rates could be swiftly identified and analyzed using RNA sequencing, potentially expediting the vaccine design process. The rapid identification and validation of neoantigens will be critical in the successful development of next-generation DNA and mRNA cancer vaccines, promising a new era of precision oncology.

### Immunosuppressive tumor microenvironment

Cancer immunotherapy faces significant challenges in eliciting effective antitumor immune responses due to the complexity of the tumor microenvironment (TME), which consists of cancer cells, stromal components like vasculature, fibroblasts, and infiltrating immune cells [218]. The interaction among these elements is prone to establish a highly immunosuppressive environment. Within the TME, most T cells become exhausted, expressing high levels of inhibitory receptors, producing fewer effector cytokines, and losing their ability to eliminate cancer cells effectively [219]. As a result, the efficacy of a single approach to cancer therapy is often limited by immunosuppressive signaling in TME, necessitating combination therapies to enhance patient outcomes [220]. In recent years, the integration of nucleic acid-based cancer vaccines with other cancer treatment modalities such as chemotherapy [220], radiation, targeted therapy, or ICB therapy has shown promising results in clinical trials. [221, 222]. For instance, neoantigen-based mRNA vaccines have demonstrated significant clinical benefits when used in conjunction with ICB and/or chemotherapy.

In a phase IIb study, mRNA-4157 combined with pembrolizumab extended recurrence-free survival among 157 patients with resected high-risk melanoma, offering an improvement over using pembrolizumab alone. [223]. Similarly, an individualized neoantigen mRNA vaccine administered alongside atezolizumab and the chemotherapy regimen mFOLFIRINOX led to the generation of substantial neoantigen-specific T cells in half of the studied cohort with resectable pancreatic ductal adenocarcinoma (PDAC). Recently, at the three-year follow-up, 6/8 patients with vaccine-induced immune responses (responders) survived, while 7/8 patients without vaccine-induced immune responses (nonresponders) experienced tumor recurrence [224]. Given the aggressive nature of PDAC—where nearly 90% of patients succumb within two years of diagnosis [108] —these findings underscore the potential of neoantigen-based cancer vaccines in combination with ICB and chemotherapy to delay or prevent the recurrence of this lethal cancer. Moreover, in patients with advanced hepatocellular carcinoma (HCC), the administration of a neoantigen-based DNA vaccine with pembrolizumab, complemented by a multityrosine kinase inhibitor, resulted in an objective response rate of 30.6%. This rate is notably higher than the approximately 17% response rate observed with pembrolizumab monotherapy, highlighting the enhanced efficacy of combining immune checkpoint inhibitors with nucleic acid-based vaccines and other cancer therapies [225, 226]. Despite these encouraging advancements, the optimal strategies for integrating ICBs, cancer drugs, and nucleic acid-based therapies remain to be fully elucidated and warrant further investigation [222].

## Conclusion

Advances in cancer therapy necessitate the integration of targeted therapies, such as CAR-T cells, antibody–drug conjugates (ADCs), and small molecules, with immune modulatory therapies, including ICIs, agonists of innate receptors, and cytokines, alongside nucleic acid-based cancer vaccines like mRNA and DNA vaccines. Targeted therapies have the potential to rapidly and precisely diminish tumor masses. Immune modulatory agents play a crucial role in alleviating the immunosuppressive environment of tumors and enhancing the infiltration of effector cells. Meanwhile, DNA and mRNA cancer vaccines are pivotal in eliciting a robust polyclonal T-cell response specifically targeting tumor cells, effectively eradicating them and eliminating residual circulating cancer cells. The swift and personalized production of DNA and mRNA vaccines is emerging as a critical element in the arsenal against human cancers. The forthcoming challenge lies in harmonizing these diverse therapeutic strategies to cure cancer effectively.

## Data Availability

Not applicable.

## Abbreviations

ABC: Amphiphilic bioresorbable copolymer
ACT: Adoptive cell therapy
AIM2: Absent in Melanoma 2
APC: Antigen-presenting cell
AR: Androgen receptor
ARCA: Anti-reverse cap analog
CAR-T: Chimeric Antigen Receptor T-cell
CCL21: C–C Motif Chemokine Ligand 21
CD4 ^+^: CD4-positive T cells
CD8 ^+^: CD8-positive T cells
CIN: Cervical intraepithelial neoplasia
CRC: Colorectal carcinoma
CRPC: Castration-resistant prostate cancer
CRT: Calreticulin
CTLA-4: Cytotoxic T-Lymphocyte-Associated Protein 4
CXCL10: C-X-C Motif Chemokine Ligand 10
DCs: Dendritic cells
DDX41: DEAD-Box Helicase 41
DNA: Deoxyribonucleic Acid
DOTAP: Dioleoyl trimethylammonium propane
DOTMA: Di-Octadecenyl trimethylammonium propane
dsRNA: Double-Stranded RNA
eIF4E: Eukaryotic Translation Initiation Factor 4E
Ep-CAM: Epithelial cell adhesion molecule
ER: Endoplasmic reticulum
GM-CSF: Granulocyte–macrophage colony-stimulating factor
HCC: Hepatocellular carcinoma
HER2: Human epidermal growth factor receptor 2
HIV-1: Human Immunodeficiency Virus Type 1
HLA-A2: Human leukocyte antigen A2
HPV: Human papillomavirus
HPLC: High-performance liquid chromatography
HSIL: High-grade squamous intraepithelial lesion
ICB: Immune checkpoint blockade
ICI: Immune checkpoint inhibitor
IFN-α: Interferon Alpha
IFN-γ: Interferon Gamma
IL-2: Interleukin 2
IL-10: Interleukin 10
IL-12: Interleukin 12
IL-23: Interleukin 23
IL-36γ: Interleukin 36 Gamma
IRAK: IL-1 receptor-associated kinase
IVT: In vitro transcription
KRAS: Kirsten Rat Sarcoma Viral Oncogene Homolog
LGP2: Laboratory of Genetics and Physiology 2
LNP: Lipid Nanoparticle
MERS: Middle East Respiratory Syndrome
MDA5: Anti-melanoma differentiation-associated gene
MDSC: Myeloid-Derived Suppressor Cell
m1Ψ: 1-Methylpseudouridine
mRNA: Messenger RNA
MHC: Major histocompatibility complex
MUC1: Mucin 1
MYD88: Myeloid differentiation primary response 88
NF-kB: Nuclear Factor Kappa-light-chain-enhancer of Activated B Cells
NK cell: Natural killer cell
NSCLC: Non-Small Cell Lung Cancer
NY-ESO-1: New York Esophageal Squamous Cell Carcinoma 1
OAS: Oligoadenylate Synthetase
ORF: Open reading frame
OX40L: OX40 Ligand
PAMPs: Pathogen-associated molecular patterns
PAP: Prostatic acid phosphatase
PFS: Progression-free survival
PD-1/PD-L1: Programmed death-1/ligand 1
PEG: Polyethylene glycol
PEI: Polyethyleneimine
PKR: Protein kinase R
PLGA: Poly(lactic-co-glycolic acid)
PRR: Pattern recognition receptor
RNA: Ribonucleic Acid
RIG-I: Retinoic acid-inducible gene I
RSV: Respiratory syncytial virus
SP: Signal peptide
ssRNA: Single-stranded RNA
STING: Stimulator of interferon genes
TAL6: Tumor-associated lymphocyte antigen 6
TAAs: Tumor-associated antigens
TAP: Transporter associated with antigen processing
TGF-beta: Transforming growth factor beta
Th1: T helper type 1
Th2: T helper type 2
TILs: Tumor-infiltrating lymphocytes
TLR7: Toll-like receptor 7
TLR8: Toll-like receptor 8
TME: Tumor microenvironment
TNBC: Triple-negative breast cancer
TRAF6: TNF receptor-associated factor 6
Tregs: Regulatory T cells
Ub: Ubiquitin
UTR: Untranslated region
CpG-ODN: CpG oligodeoxynucleotide
